# Consensus Minimal Dataset for Pediatric Emergency Medicine in Switzerland

**DOI:** 10.1097/PEC.0000000000002841

**Published:** 2022-09-11

**Authors:** Alice C. Wismer, Milenko Rakic, Claudia E. Kuehni, Manon Jaboyedoff, Fabrizio Romano, Matthias V. Kopp, Julia Brandenberger, Georg Staubli, Kristina Keitel

**Affiliations:** From the ∗ Pediatric Emergency Medicine, Department of Pediatrics, Inselspital, Bern University Hospital, University of Bern, Switzerland; † Institute of Social and Preventive Medicine (ISPM), University of Bern, Bern, Switzerland; ‡ Service of Pediatrics, Department Women-Mother-Child, Lausanne University Hospital and University of Lausanne, Lausanne, Switzerland; § Department of Pediatrics, Inselspital, Bern University Hospital, University of Bern, Switzerland; ∥ Division of Pediatric Emergency Medicine, Hospital for Sick Children, Toronto, Ontario, Canada; ¶ Department of Pediatric Emergency Medicine, Childrens' University Hospital Zurich, Zurich, Switzerland.

**Keywords:** electronic health records and systems, registries, common data model, data harmonization

## Abstract

**Objectives:**

Standardized, harmonized data sets generated through routine clinical and administrative documentation can greatly accelerate the generation of evidence to improve patient care. The objective of this study was to define a pediatric emergency medicine (PEM) minimal dataset for Switzerland (Swiss PEM minimal dataset) and to contribute a subspecialty module to a national pediatric data harmonization process (SwissPedData).

**Methods:**

We completed a modified Delphi survey, inviting experts from all major Swiss pediatric emergency departments (PEDs).

**Results:**

Twelve experts from 10 Swiss PEDs, through 3 Delphi survey rounds and a moderated e-mail discussion, suggested a subspecialty module for PEM to complement the newly developed SwissPedData main common data model (CDM). The PEM subspecialty CDM contains 28 common data elements (CDEs) specific to PEM. Additional CDEs cover PEM-specific admission processes (type of arrival), timestamps (time of death), greater details on investigations and treatments received at the PED, and PEM procedures (eg, procedural sedation). In addition to the 28 CDEs specific to PEM, 43 items from the SwissPedData main CDM were selected to create a Swiss PEM minimal dataset. The final Swiss PEM minimal dataset was similar in scope and content to the registry of the Pediatric Emergency Care Applied Research Network.

**Conclusions:**

A practical minimal dataset for PEM in Switzerland was developed through recognized consensus methodology. The Swiss PEM minimal dataset developed by Swiss PEM experts will facilitate international data sharing for PEM research and quality improvement projects.

Standardized, harmonized datasets generated through routine clinical and administrative documentation can significantly accelerate evidence generation to improve patient care.^1^ To date, evidence for pediatric care largely stems from research-based data collection, which may be fragmented and biased by research funding priorities.^2^ More systematic and inclusive approaches to data collection are largely missing, although they would be particularly important for pediatrics. Children represent a smaller patient group compared with adults; relevant events, such as serious emergency events, are often rare.^3^ Disease patterns are more heterogeneous because of differences in epidemiology and clinical presentation across the pediatric age spectrum.^3,4^ To reach adequate sample sizes, harmonized datasets across multiple institutions are needed.^3^

In pediatric emergency medicine (PEM), this need for cross-institutional collaboration has been recognized and has led to the formation of international, collaborative clinical research networks.^5,6^ Examples include the Pediatric Emergency Care Applied Research Network (PECARN),^7^ the Pediatric Emergency Research of Canada (PERC),^8^ Pediatric Emergency Research in the UK and Ireland (PERUKI),^9^ Pediatric Research in Emergency Departments International Collaborative (PREDICT),^10^ The European Pediatric Emergency Medicine Research Network (REPEM), and the umbrella network Pediatric Emergency Research Network (PERN).^6,11^ The PECARN network has defined a PEM minimal dataset generated from routine health care data.^12,13^

In Switzerland, electronic health records (EHRs) and related documentation of clinical data are neither harmonized nor interoperable. The Swiss Federal Government thus initiated the Swiss Personalized Health Network (SPHN) initiative to create a nationwide, interoperable data platform for health-relevant data.^14^ Under the umbrella of SPHN, a pediatric-specific data harmonization project, the SwissPedData project, aimed at developing a common data model (CDM) across Swiss pediatric hospitals.^15^ SwissPedData involved a multistage consensus process including pediatric providers from all Swiss pediatric tertiary care hospitals to reach agreement on 1) a list of common data elements (CDEs) for SwissPedData, 2) standardized answer format for each CDE, and 3) a classification of each CDE as either mandatory, recommended, or optional.

The first version of the SwissPedData CDM consists of a main module (general pediatrics and data elements relevant for all subspecialties) and 10 subspecialty-specific modules (cardiology, endocrinology, gastroenterology, immunology-allergology, infectious diseases, metabolic diseases, nephrology, neurology, pulmonology, and rheumatology). In each module, CDEs are organized into 9 categories (care site, demographics, medical history, physical examination, clinical scores, investigations, diagnosis, treatment, and equipment/procedures).

The overall goal of the consensus process described in this article was to contribute a PEM subspecialty module to the SwissPedData CDM and to define a minimal dataset for PEM in Switzerland (analogous to the PECARN minimal dataset). This minimal dataset would draw data elements from the SwissPedData main module (already agreed on ahead of the PEM subspecialty project) in addition to the PEM subspecialty data elements (Fig. 1).

**FIGURE 1:**
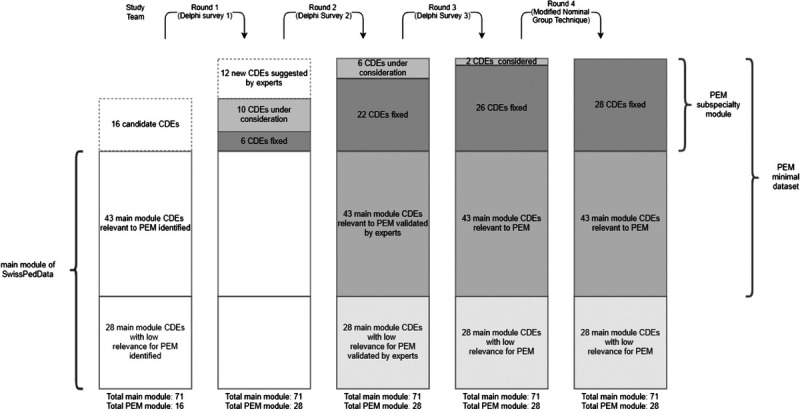
Consensus process for PEM subspecialty module and definition of PEM minimal dataset (Swiss PEM minimal dataset).

## METHODS

We performed a modified 3-stage Delphi method followed by a modified nominal group method between May and October 2020. Delphi processes are used to reach consensus on complex issues through an iterative, structured group communication process. Aggregated group answers from previous rounds are provided with each new questionnaire, and the experts being questioned are able to reconsider their judgments on this basis, revising them where appropriate.^16^ The study was coordinated by the Department of Pediatric Emergency Medicine of the University Hospital Inselspital, Bern, Switzerland, in collaboration with the SwissPedData project and the Swiss Pediatric Emergency Medicine Network (Pediatric Emergency Medicine Switzerland [PEMS]).

## PARTICIPANT SELECTION

Pediatric emergency physicians from all PEM departments in Switzerland were identified through the PEMS network and invited to participate in the consensus process. Invitations were distributed via e-mail by the head of department at each site. All the volunteering experts were invited for the survey without an additional selection process.

## DATASET

The Swiss PEM minimal dataset (“*Swiss PEM minimal dataset”*) consists of a selection of CDEs from the SwissPedData main module and of additional, consensus-based CDEs of the PEM subspecialty module (Fig. 1).

### SwissPedData Main Module—Selection of PEM-Relevant CDEs

The SwissPedData main module was developed before the start of the PEM consensus process described here. The process and content are published elsewhere.^15^ The final main module is shown in Supplementary Table 2, http://links.lww.com/PEC/B24. To define the Swiss PEM minimal dataset, data elements relevant to PEM were preselected by the coordinating team. Participants were asked to validate this selection during the survey.

### PEM Subspecialty Module—Selection of Candidate CDEs

The data categories (care site, demographics, medical history, physical examination, clinical scores, investigations, diagnosis, treatment, and equipment/procedures) were predefined by the SwissPedData project.

Before the first survey, the coordinating team suggested possible CDEs to be considered for inclusion for each category for the PEM module. This was done based on the PECARN minimal dataset and on clinical data elements routinely documented in PEM. By suggesting CDE candidates, emphasis was put on clinical significance and feasibility of collection within the existing Swiss PEM workflows and electronic documentation.

## CONSENSUS PROCESS

We used a modified online Delphi process as a group consensus strategy^16^ (Fig. 1). The same experts were interrogated in several rounds. The classical Delphi survey rounds were complemented by a modified nominal group method step, which was implemented through a moderated e-mail discussion to reach final consensus. The consensus-finding process aimed to reach agreement on 1) a list of CDEs for PEM, 2) standardized answer format for each CDE, and 3) a classification of each CDE as either mandatory, recommended, or optional. Each expert was asked to vote for inclusion or exclusion of each candidate CDE. When opting for inclusion of a CDE, experts were further asked to classify the CDE as mandatory, recommended, or optional, using the following definitions from the SwissPedData project:

- Mandatory if available: the CDE should be collected for each child seen in the PDE if the information is relevant and available.
- Recommended if available: the CDE should be collected on a voluntary basis.
- Optional: The CDE should be collected for specific research projects only.

Based on published reports from similar clinical modified Delphi processes,^16,17^ we set the level of agreement at 75% or higher. If no consensus on parts 2 to 3 (answer format and level of requirement) could be reached during a survey round, the most frequently selected answer from the previous round was presented. Experts were then asked whether they agreed with this answer choice.

Online surveys were programmed using the SurveyMonkey software (SurveyMonkey, Inc, San Mateo, CA) and analyzed using Microsoft Excel, Version 2010 (Microsoft Corp, Redmond, WA).

### Round 1—Delphi Survey 1

During round 1, experts received a general introduction to the SwissPedData CDM, including its purpose and format. The SwissPedData main module was presented along with the candidate PEM CDEs suggested by the coordinating team. Each expert was asked to vote for inclusion or exclusion of each candidate CDE and to suggest any additional CDEs. When opting for inclusion of a CDE, experts were further asked to classify the CDE as “mandatory”, “recommended”, or “optional”. Experts were asked to suggest answer formats for newly suggested CDEs.

### Round 2—Delphi Survey 2

During round 2, we asked experts to vote on PEM CDEs (CDEs proposed by the coordinating team and additional CDEs suggested during round 1, if applicable). In this round, experts were also asked to validate the selection of data elements relevant to a Swiss PEM minimal dataset from the SwissPedData main module.

### Round 3—Delphi Survey 3

As in round 2, experts were again asked to vote on PEM subspecialty CDEs during round 3.

### Round 4—Modified Nominal Group Method

Analogous to a modified nominal group method, we implemented a moderated e-mail discussion to reach consensus on the outstanding data elements after round 3. In these e-mail discussions, reasons for previous choices were exchanged during this discussion, and experts were asked to revote after the discussion.

## RESULTS

### Participants

A total of 12 experts were recruited from 10 Swiss hospitals (Aarau, Baden, Basel, Bellinzona, Bern, Geneva, Lucerne, St. Gallen, and Zurich [2 hospitals]) via the PEMS network for the survey (Supplementary Table 1, http://links.lww.com/PEC/B24). All experts participated during all 3 rounds.

### Consensus Process for PEM CDM

The coordinating team suggested 16 candidate CDEs for the PEM subspecialty module. During round 1, experts proposed 12 additional CDEs (Fig. 1). All 28 PEM subspecialty CDEs were finally included into the CDM. During round 2 and 3, agreement was reached on the level of requirement for 26 of the 28 PEM subspecialty elements. For the CDEs “citizenship/type of permit” and “date and time of scheduled last administration”, no consensus could be reached after 3 survey rounds. These CDEs were hence discussed in a moderated e-mail discussion. For “citizenship/type of permit” some experts were concerned about discrimination against minority groups if the CDE was collected. Other experts argued that, to the contrary, collection of this information would be vital to improve equitable care for minority groups. After discussion, consensus was reached that the CDE should be “recommended if available”. For the CDE “date and time of scheduled last administration”, some experts misread the most common answer. After clarification of this misunderstanding, consensus could be reached on “recommended if available”. The final PEM subspecialty module is summarized in Table 1.

**TABLE 1:** Pediatric Emergency Medicine Subspecialty CDM for SwissPedData

Data Category	PEM-Specific CDE	Importance
1. Care site	Method of arrival	M
	Date and time of triage	M
2. Demographics	Citizenship/type of permit	R
3. Medical history	Medical history	M
	Vaccinations	M
4. Physical examination	Pain scale, type	M
	Pain scale, value	M
	Capillary refill time	M
5. Clinical scores	*no additional CDEs suggested to main module*	
6. Investigations	Laboratory test performed (yes/no)	M
	Type of laboratory test	M
	Urine collection method	M
	Other diagnostic test	M
	Specialist consultation in the ED	R
7. Diagnosis	Time of death	M
8. Treatment	Outpatient medications	M
	Route of administration	M
	Date and time of first administration	R
	Date and time of scheduled last administration	R
	Date and time of effective last administration	M
	Frequency of administration	M
	Dose	M
	Dose unit	M
	Supplemental O_2_: type of application	M
9. Equipment and procedures	Equipment: time of insertion	M
	Equipment: time of withdrawal	M
	ED procedure	M
	Procedural sedation (yes/no)	M
	Type of procedural sedation	M

Please refer to (Supplementary Table 3, http://links.lww.com/PEC/B24) for format details.

ED indicates emergency department; M, mandatory; O_2_, oxygen; R, recommended.

### Swiss PEM Minimal Dataset

As part of the overall consensus process, the group also defined a standalone Swiss PEM minimal dataset. This minimal dataset is composed of the PEM subspecialty module as well as CDEs relevant to PEM from the main module (Fig. 1). The coordinating team suggested 43 of the 71 CDEs from the main module as relevant to PEM (Fig. 1, Table 2). This selection was validated by the expert group during round 2 (Fig. 1). The final PEM minimal dataset is summarized in Table 1.

**TABLE 2:** Swiss PEM Minimal Dataset Composed of CDEs From the SwissPedData Main Module (Purple) and PEM Subspecialty Module (Blue)

	1. Care Site	2. Demographics	3. Medical History	4. Physical Examination	5. Clinical Scores	6. Investigations	7. Diagnosis	8. Treatment	9. Equipment and Procedures
CDEs relevant to PEM selected from the SwissPedData main module	- Type of admission- Type of arrival - Care Handling Type - Visit-start date and time - Visit-end date and time - Date and time of admission - Discharge destination – Hospital – Department - Unit	- Patient date and time of birth - Patient administrative sex - Address (postal code)	- Reason for consultation / for admission - Drug allergies - Documented food allergies	- Heart rate - Systolic blood pressure - Diastolic blood pressure - Respiratory rate - Oxygen saturation – Temperature – Weight - Height
PEM-specific additional CDEs proposed by coordinating team (PEM subspecialty module)	- Method of Arrival - Date and time of Triage	- Citizenship/type of permit		- Pain scale, type - Pain scale, value
PEM-specific additional CDEs proposed by experts in Round 1 (PEM subspecialty module)			- Past medical history - Vaccinations	- Capillary refill time
- Triage scale (ED), type - Triage scale (ED), value - AVPU score - Glasgow Coma Scale	- Type of imaging study (detailed) - Date and time of imaging study - Indication for the imaging study	- Diagnosis - Date of diagnosis - Cause of death - Date of death	- Inpatient medications (including drug name, route of administration, dosing, duration) - Discharge medications (including drug name, route of administration, dosing, duration) - Adverse events - Supplemental O_2_: Date and time of start - Supplemental O_2_: Date and time of discontinuation	- Equipment type - Equipment: date of insertion - Equipment: date of withdrawal
	- Laboratory test performed (yes/no) - Type of laboratory test - Urine collection method - Other diagnostic test - Specialist consultation in the ED	- Time of death	- Outpatient medications, including:	- Equipment: time of insertion - Equipment: time of withdrawal - Emergency department procedure
			- Route of administration - Date and time of first administration - Date and time of scheduled last administration - Date and time of effective last administration - Frequency of administration - Dose - Dose unit- -Supplemental O_2_: Type of application	- Procedural sedation (yes/no) - Type of procedural sedation

Please refer to (Supplementary Table 3, http://links.lww.com/PEC/B24) for format details.

AVPU indicates alert, voice, pain, unresponsive.

## DISCUSSION

Through a consensus process, experts from 10 PEDs in Switzerland developed a subspecialty module for PEM to complement the newly developed SwissPedData CDM. The resulting Swiss PEM minimal dataset includes 43 items from the SwissPedData main module and 28 items specific for PEM. Additional CDEs cover PEM-specific admission processes (type of arrival), timestamps (time of death), and greater details on investigations and treatments received in the emergency department as well as PEM-specific procedures (eg, procedural sedation).

### Comparison With Other PEM CDMs/Minimal Datasets

The PECARN network developed and implemented an electronic health record-based registry with harmonized data elements from 7 academic emergency departments. The registry encompasses 176 distinct CDEs including demographics, encounter characteristics, timestamps, vital signs, clinical scores, clinical care orders, results, medications, coded diagnoses and procedures, and free-text narratives from the entire PED encounter. A working group of epidemiologists, quality improvement scientists, clinicians, informaticists, data analysts, biostatisticians, and research coordinators was established to define the data elements. The PECARN CDEs are similar to the CDEs proposed through our consensus process, which makes the 2 CDMs compatible and could allow data sharing. Additional PECARN data elements not included in the Swiss PEM minimal dataset are ethnicity, race, provider information, insurance details, and an asthma score. Race and ethnicity are not routinely collected in Swiss EMRs. The PECARN data model captures PED-specific processes, for example, through inclusion of timestamp CDEs to understand the sequence of events. Although, to our knowledge, the original CDM was not consensus-based, the PECARN registry is very comprehensive and already implemented across several PED sites.

### Strengths and Limitations

The Delphi expert group in this study was composed of 12 practicing physicians from 10 different PEDs. The group size was relatively small but covered all major PEDs in Switzerland and represented various levels of academic experience and seniority. The optimal panel size for a Delphi method is unknown and differs depending on the aim of the study and available resources.^16^ Selection bias remains a concern because all experts volunteered to participate. We only invited physicians as experts in this consensus process. Physicians-in-training (residents) were underrepresented, and other professions, such as nursing and administrative staff, were not included. The German-speaking part of Switzerland was overrepresented compared with other areas.

We used a modified Delphi process. The online survey and e-mail discussion format allowed participation of PEM experts with high clinical workload. The SARS-CoV-2 pandemic did not allow for physical meetings, which would have allowed for a more in-depth review and discussion. We also recognize that results from a consensus process do not systematically assess evidence.^18^ Suggestions for data elements were generated from a review of existing datasets and expert experience. In contrast to the PECARN process, we did not systematically examine existing EHRs, which could have assured that all important CDEs are included. One strength of this study was a high level of agreement after 3 consent rounds (26 of 28 items), suggesting clear priority elements.

### Outlook

The consensus-based definition of the Swiss PEM minimal dataset described here only represents a 1st step toward a common harmonized Swiss PEM database. Important implementation-related steps remain to be defined and implemented within the SPHN project. A particular challenge for implementation in Switzerland is the heterogeneity of EHRs. Ethical and data safety remain regulated at the cantonal level in Switzerland and would need to be harmonized on a federal level. Some of the vendors of EHRs implemented in Switzerland are already included in the PECARN registry. In addition to a national approach to data harmonization, an international process could be considered. This would further improve the size of the dataset and allow comparisons of outcomes across different health systems. Standardization of data elements across countries may also require the description and/or standardization of ED workflows across providers. Standardized data terminologies are still lacking for most PEM data domains, which may require additional manual steps to identify CDE values. For PEM, the inclusion of timestamps is particularly relevant, which adds complexity to the data model.

Potential use of a Swiss PEM minimal dataset include not only PEM-relevant research questions but also PEM-quality metrics, including equity aspects. Such performance metrics and data on disparities in pediatric emergency care have begun to be implemented in Europe.

## CONCLUSIONS

Pediatric emergency medicine experts representing all large PEDs in Switzerland reached agreement on the first Swiss national PEM minimal dataset for standardized data collection on PEDs. The final Swiss PEM minimal dataset proposed is very similar to an existing American PEM registry in content and scope. The proposed data model will be integrated into an overall Swiss project to harmonize data capturing for pediatric patients in routine care. It will be the prerequisite for national and international standardization of data elements to promote evidence in pediatric care.

## Supplementary Material

SUPPLEMENTARY MATERIAL
