# Supplementary material for: Consensus Minimal Dataset for Pediatric Emergency Medicine in Switzerland
Source: Pediatr Emerg Care. 2022 Sep 11;38(10):511–6. doi: 10.1097/PEC.0000000000002841 (PMC9555753; doi:10.1097/PEC.0000000000002841)
Supplement: SUPPLEMENTARY MATERIAL [file pcare-38-511-s001.docx]

# Supplemental Digital Content

|  | Experts (N=12) |
| --- | --- |
| Gender | 7 Female (58%)  5 Male (42%) |
| Swiss Region | 10 from German-speaking region (83%)  1 from French-speaking region (8%)  1 from Italian-speaking region (8%) |
| Function | 4 Physicians with leadership position (33%)  7 Senior Physicians (58%)  1 Resident Physicians (8%) |
| Years of Experience in Pediatric Emergency Medicine | Median: 4.5 years; IQR: 6.5 years  6 experts with 1-5 years of experience (50%)  4 experts with 5-10 years of experience (33%)  2 experts with 11-20 years of experience (17%) |
| PEM board certification | 9 experts with PEM subspecialty board certification (75%) |
| Scholarly activity | 5 experts without research activity (42%)  5 experts participating in research projects (42%)  2 experts leading research projects (17%) |

Supplementary Table 1 Characteristics of Experts
IQR = Interquartile Range; PEM = Pediatric Emergency Medicine

| **Category** | **Common Data Element** | **Format** | **Standardized response options** | **Importance** | **Comment / Description** |
| --- | --- | --- | --- | --- | --- |
| **Care site** | **Type of admission** | standardized options | Elective admission  Emergency admission | Mandatory |  |
|  | **Type of arrival** | standardized options | Transfer from outside hospital  Transfer from outside emergency department  Referral from pediatrician/general practitioner  Walk-in Other | Mandatory |  |
|  | **Care Handling Type** | standardized options | Inpatient  Outpatient | Mandatory |  |
|  | **Visit-start date and time** | date and time | YYYY-MM-DD hh:mm:ss | Mandatory | Date and time at which the interaction between individual and the care provider institution started |
|  | **Visit-end date and time** | date and time | YYYY-MM-DD hh:mm:ss | Mandatory | Date and time at which the interaction between individual and the care provider institution stopped |
|  | **Date and time of admission** | date and time | YYYY-MM-DD hh:mm:ss | Mandatory | Date and time of patient's inpatient admission to the care provider institution |
|  | **Discharge destination** | standardized options | Home  Other hospital  Institution  Other | Mandatory | Discharge destination |
|  | Follow-up after discharge / consultation | standardized options | General pediatrician  General practitioner  Subspecialist  Nurse  None | Mandatory | Scheduled follow-up at discharge |
|  | Translator used | standardized options | Yes  No  Unknown | Recommended | Translator used for communication between patient and healthcare team |
|  | **Hospital** | standardized options | See comments | Mandatory | Standardized response options will be the name of participating children’s hospitals |
|  | **Department** | standardized options | See comments | Mandatory | Standardized response options will be the name of departments of participating children’s hospitals |
|  | **Unit** | standardized options | See comments | Mandatory | Standardized response options will be the name of units of participating children’s hospitals |
| **Demographics** | **Patient date and time of birth** | date and time | YYYY-MM-DD hh:mm:ss | Mandatory | Date and time of birth of the patient |
|  | Country of birth | standardized options | Swiss Federal Statistical Office: ISO code of the country of origin | Mandatory | Country of birth of the patient |
|  | Place of birth (CH) | number | Postal code | Mandatory | Municipality of birth of the patient if in Switzerland, coded by postal codes. |
|  | **Patient administrative gender** | standardized options | Male  Female  Other | Mandatory |  |
|  | **Address (postal code)** | number | Postal code | Mandatory | Current address of the patient, coded by postal codes. Exact address should also be recorded |
|  | Nationality | standardized options | Swiss Federal Statistical Office: ISO code of the country of origin | Mandatory | Current nationality of the patient |
|  | Date of immigration | date | YYYY-MM-DD | Mandatory | Date of first immigration to Switzerland if born abroad |
| **Medical history** | **Reason for consultation /  for admission** | free text |  | Mandatory | Main reason for consultation or for admission. Standard classification not defined. |
|  | Use of complementary medicine | yes/no |  | Optional | Patient treated with complementary medicine at home or in hospital |
|  | Birth weight | number |  | Mandatory | Weight at birth in kg |
|  | Birth length | number |  | Mandatory | Length at birth in cm |
|  | Birth's head circumference | number |  | Mandatory | Head circumference at birth in cm |
|  | Delivery mode | standardized options | Caesarean section  Instrumental vaginal delivery Spontaneous vaginal delivery | Mandatory | Birth delivery mode |
|  | Gestational age | number |  | Mandatory | Post-menstrual age at birth in week and days |
|  | Apgar score 1 min | number |  | Recommended | Apgar score 1 min after birth |
|  | Apgar score 5 min | number |  | Recommended | Apgar score 5 min after birth |
|  | Apgar score 10 min | number |  | Recommended | Apgar score 10 min after birth |
|  | Mother's year of birth | number |  | Mandatory | Year of birth of the mother |
|  | Father's year of birth | number |  | Mandatory | Year of birth of the father |
|  | Year(s) of birth of sibling(s) | number |  | Mandatory | Year of birth of sibling(s) if any |
|  | **Drug allergies** | standardized options | International nonproprietary name of drug | Mandatory | Known drug allergies |
|  | **Documented food allergies** | yes/no |  | Mandatory | Presence of any documented food allergy |
| **Physical examination** | **Heart rate** | number |  | Mandatory | Heart rate in beats per minute |
|  | **Systolic blood pressure** | number |  | Mandatory | Value of the systolic blood pressure in mmHg |
|  | **Diastolic blood pressure** | number |  | Mandatory | Value of the diastolic blood pressure in mmHg |
|  | **Respiratory rate** | number |  | Mandatory | Respiratory rate in breaths per minute |
|  | **Oxygen saturation** | number |  | Mandatory | Measured oxygen saturation in % |
|  | **Temperature** | number |  | Mandatory | Measured temperature of the patient in Celsius degrees |
|  | **Weight** | number |  | Mandatory | Measured weight of the patient in kg |
|  | **Height** | number |  | Mandatory | Measured height of the patient in cm |
|  | Head circumference | number |  | Mandatory | Measured head circumference of the patient in cm |
| **Clinical scores** | **Triage scale (ED), type** | standardized options | Australasian Triage Scale  Canadian Triage Scale  Other | Mandatory | Name of the triage scale used |
|  | **Triage scale (ED), value** | number |  | Mandatory | Value of the triage scale |
|  | **AVPU score** | standardized options | Alert Voice Pain  Unresponsive | Mandatory |  |
|  | **Glasgow Coma Scale** | number |  | Mandatory |  |
| **Investigations** | **Type of imaging study (detailed)** | standardized options | See comments | Mandatory | Standard classification to be defined |
|  | **Date and time of imaging study** | date and time | YYYY-MM-DD hh:mm:ss | Mandatory | Date and time of the radiological study |
|  | Radiation dose | number |  | Mandatory | If applicable, dose of radiation in mSv |
|  | **Indication for imaging study** | free text |  | Mandatory |  |
| **Diagnosis** | **Diagnosis** | See comments | See comments | Mandatory | Inpatients diagnosis are ICD10 coded and outpatients diagnosis are free text. |
|  | **Date of diagnosis** | date | YYYY-MM-DD | Mandatory |  |
|  | **Cause of death** | See comments | See comments | Mandatory | Standard classification to be defined |
|  | **Date of death** | date | YYYY-MM-DD | Mandatory |  |
| **Treatment** | **Inpatient medication** | standardized options | International non-proprietary name | Mandatory | Name of the drug(s) received as inpatient |
|  | **Discharge medications** | standardized options | International non-proprietary name | Mandatory | Name of the drug(s) prescribed at discharge |
|  | Route of administration | standardized options | Oral  Intravenous  Subcutaneous  Intramuscular Intrathecal  Rectal  Inhalation  Cutaneous  Ocular  Nasal  Otic  Other | Mandatory |  |
|  | Date and time of first administration | date and time | YYYY-MM-DD hh:mm:ss | Mandatory | Time of first administration of the drug |
|  | Date and time of last administration | date and time | YYYY-MM-DD hh:mm:ss | Mandatory | Time of last administration of the drug |
|  | Frequency of administration | number |  | Mandatory | Number of administrations per 24 hours |
|  | Dose | number |  | Mandatory | Dose given at each administration of the drug |
|  | Dose unit | standardized options |  | Mandatory | List of possible units to be defined |
|  | Reason for discontinuation of treatment | standardized options | Recovery  Change to another medication  No effect observable  Adverse events  Reducing polypharmacy  Other | Mandatory | Reason why a treatment is stopped |
|  | **Adverse events** | standardized options | MedDRA classification | Mandatory | MedDRA: Medical Dictionary for Regulatory Activities |
|  | **Supplemental O2: date and time of start** | date and time | YYYY-MM-DD hh:mm:ss | Mandatory |  |
|  | **Supplemental O2: date and time of discontinuation** | date and time | YYYY-MM-DD hh:mm:ss | Mandatory |  |
|  | Supportive services: Type | standardized options | Physiotherapy  Ergotherapy  Social service  Other | Mandatory |  |
| **Equipment and procedures** | **Equipment type** | standardized options | See comments | Mandatory | Standard classification to be defined |
|  | **Equipment: date of insertion** | date | YYYY-MM-DD | Mandatory |  |
|  | **Equipment: date of withdrawal** | date | YYYY-MM-DD | Mandatory |  |

Supplementary Table 2 SwissPedData Main Data Module. Variables that were selected by experts for the Swiss PEM minimal dataset are bold.

AVPU = Alert, Voice, Pain, Unresponsive; CH = Switzerland; ED = Emergency Department; ICD = International Statistical Classification of Diseases and Related Health Problems; ISO = International Organization for Standardization; MedDRA = Medical Dictionary for Regulatory Activities; O2 = Oxygen

| Category | Common Data Element | Format | Standardized response options | Importance | Comment / Description | Variable suggested by | Consensus first round | Consensus secound round Data requirement accepted |
| --- | --- | --- | --- | --- | --- | --- | --- | --- |
| Care site | **Method of Arrival** | standardized options | 1 Air ambulance 2 Road ambulance 3 Own transport (walk-in) 4 Other 88 Unknown | Mandatory | How did the patient arrive at the ED | Coordinating team | Mandatory (75%) | Yes (first round) |
|  | **Date and time of Triage** | date | YYYY-MM-DD hh:mm:ss | Mandatory | Date and time of ED Triage | Coordinating team | Mandatory (92%) | Yes (first round) |
| Demographics | **Citizenship/type of permit** | standardized options | 1 Citizen 2 B 3 C 4 Ci 5 G 6 L 7 F 8 N 9 S 10 Undocumented 88 Unknown | Recommended | Citizenship or if immigrant, type of residency permit | Coordinating team | Recommended (50%) | No (67% agree - secound round) 🡪 MNGM |
| Medical history | **Past medical history** | free text |  | Mandatory | Prior surgical history, interventions, chronic disease etc. | Experts | Mandatory (58%) | Yes (100% - secound round) |
|  | **Vaccinations** | standardized options | 1 Yes, according to current national immunization schedule 2 Yes, but incompletely/not according to current national immunization schedule 3 No | Mandatory | Is the child vaccinated | Experts | Mandatory (92%) | Yes (first round) |
| Physical examination | **Pain scale, type** | standardized options | 1 Face, Legs, Activity, Cry, Consolability Scale (Revised) (FLACC-R e.g. KUS/KUSS) 2 Faces Pain Scale - (Revised) (FPS-R e.g. Bieri, Hicks) 3 Numeric Pain Raiting Scale (NRS-10 e.g. VAS) 4 Children's Hospital of Eastern Ontario Pain Scale (CHEOPS) 5 Neonatal Infant Pain Scale (NIPS) 6 Non-Communicating Children's Pain Checklist - (Revised) (NCCPC-R) 7 Alder Hay Triage Pain Score (AHTPS) 8 Other | Mandatory | Name of the pain scale used | Coordinating team | Mandatory (58%) | Yes (92% - secound round) |
|  | **Pain scale, value** | number | (Number) | Mandatory | Value of the pain scale | Coordinating team | Mandatory (83%) | Yes (first round) |
|  | **Capillary refill time** | number | Number in secounds | Mandatory | Microcirculation | Experts | Mandatory (92%) | Yes (first round) |
| Clinical scores | **NO ADDITIONAL** |  | **CDEs SUGGESTED** | **TO MAIN** | **MODULE** |  |  |  |
| Investigations | **Laboratory tests** | standardized options | 1 Yes 2 No 88 Unknown | Mandatory | Laboratory tests performed | Coordinating team | Mandatory (92%) | Yes (first round) |
|  | **If laboratory tests, type** | standardized options | 1 Blood 2 Urine 3 Cerebrospinal fluid 4 Joint effusion 5 Respiratory sample 6 Stool 7 Other | Mandatory | Type of laboratory tests | Coordinating team | Mandatory (92%) | Yes (first round) |
|  | **If urine tests,  urine collection method** | standardized options | 1 Urethral catheterization 2 Clean catch void 3 Urine collection bag 4 Mid-stream urine 5 Suprapubic aspiration 88 Unknown | Mandatory | Applied urine collection method | Coordinating team | Mandatory (75%) | Yes (first round) |
|  | **Other diagnostic tests** | standardized options | 1 ECG 2 EEG 3 Two-channels raw real-time bed-side EEG 4 Transthoracal echocardiography (TTE) 5 Other | Mandatory | Other diagnostic tests performed | Coordinating team | Mandatory (58%) | Yes (100% - secound round) |
|  | **Specialist consultation in the ED** | standardized options | 1 None 2 Cadiology 3 Dermatology 4 Endocrinology 5 Gastroenterology 6 Gynaecology 7 Infectious Diseases 8 Nephrology 9 Neurology 10 Oncology 11 Ophthalmology 12 Surgery/Orthopaedics 13 Otorhinolaryngology 14 Pulmonology 15 Rheumatology 16 Allergology 17 Psychology/psychiatry 18 Other | Recommended | Consultation of a specialist | Coordinating team | Recommended (50%) | Yes (100% - secound round) |
| Diagnosis | **Time of death** | time | hh:mm:ss | Mandatory |  | Coordinating team | Mandatory (92%) | Yes (first round) |
| Treatment | **Outpatient medications (administered at care site)** | standardized options | International non-proprietary name | Mandatory | Name of the drug(s) received as outpatient | Coordinating team | Mandatory (83%) | Yes (first round) |
|  | **..Route of administration** | standardized options |  | Mandatory | Route of administration of the drug (IV, PO…) | Experts | Mandatory (75%) | Yes (first round) |
|  | **Date and time of first administration** | date and time | YYYY-MM-DD hh:mm:ss | Recommended | Time of first administration of the drug | Experts | Recommended (50%) | Yes (92% - secound round) |
|  | **Date and time of scheduled last administration** | date and time | YYYY-MM-DD hh:mm:ss | Recommended | Time of last planned administration of the drug when prescribed | Experts | Recommended (33%) | No (58% agree - secound round) 🡪 MNGM |
|  | **Date and time of effective last administration** | date and time | YYYY-MM-DD hh:mm:ss | Mandatory | Time of last administration in-hospital | Experts | Mandatory (50%) | Yes (83% - secound round) |
|  | **Frequency of administration** | number |  | Mandatory | Number of administration per 24 hours | Experts | Mandatory (67%) | Yes (100% - secound round) |
|  | **Dose** | number |  | Mandatory | Dose given at each administration of the drug | Experts | Mandatory (75%) | Yes (first round) |
|  | **Dose unit** | standardized options |  | Mandatory | Unit of the dose | Experts | Mandatory (75%) | Yes (first round) |
|  | **Supplemental O2: Type of application** | standardized options | 1 Nasal prongs 2 Mask 3 High-flow 4 Ventilated (ET tube, NIV) | Mandatory | Type of oxygen application | Coordinating team | Mandatory (75%) | Yes (first round) |
| Equipment and procedures | **Equipment time of insertion** | time | hh:mm:ss | Mandatory | Time of insertion of the equipment | Coordinating team | Mandatory (75%) | Yes (first round) |
|  | **Equipment time of withdrawal** | time | hh:mm:ss | Mandatory | Time of withdrawal of the equipment | Coordinating team | Mandatory (75%) | Yes (first round) |
|  | **ED procedure** | standardized options | 1 Peripheral vein catheter 2 Wound repair 3 Abscess incision and drainage 4 Noninvasive ventilation 5 Cardiopulmonary resuscitation (CPR) 6 Foreign body removal eye 7 Foreign body removal nose 8 Foreign body removal ear 9 Foreign body removal other 10 Gastrostomy tube replacement 11 Reduction of an hernia 12 Diagnostic puncture of joint 13 Depirdement of burn wound 14 Intra-osseous access 15 Urine catheter 16 Central vein catheter 17 Pleural puncture 18 Lumbar puncture 19 Reduction of fracture/dislocated joint 20 Splint 21 Cast 22 Regional anaesthesia 23 Supraventricular tachycardia conversion 24 Other | Mandatory | Permitted procedures on the Emergency Department | Coordinating team | Mandatory (75%) | Yes (first round) |
|  | **Procedural sedation** | yes/no |  | Mandatory | Was there the need for procedurale Sedation | Experts | Mandatory (100%) | Yes (first round) |
|  | **If yes, procedural sedation type** | standardized options | 1 Mild/moderate sedation (Midazolam oral/nasal/rectal, Dexmedetomidine, Nitrous Oxide) 2 Dissotiative Sedation (Ketamin IV/IM) 3 Deep Sedation (Propofol IV) 4 Other | Mandatory | What type of procedural sedation was conducted | Experts | Mandatory (100%) | Yes (first round) |

Supplementary Table 3: Details of final PEM subspecialty module

CDE = Common Data Element; ECG = Electrocardiogram; ED = Emergency department; EEG = Electroencephalogram; ET = endotracheal; IM = intra muscular; IV = intra venous; KUS/ KUSS = Kindliche Unbehangens- und Schmerzskala (engl. Childish Uneasiness and Pain Scale); MNGM = Modified Nominal Group Method; NIV = non-invasive ventilation; O2 = Oxygen; PEM = Pediatric emergency medicine; PO = per oral; VAS = Visual Analogue Scale
